# [Expression of Concern] S100A8-targeting siRNA enhances arsenic trioxide-induced myeloid leukemia cell death by down-regulating autophagy

**DOI:** 10.3892/ijmm.2025.5622

**Published:** 2025-09-01

**Authors:** Liangchun Yang, Minghua Yang, Hong Zhang, Zhuo Wang, Yan Yu, Min Xie, Mingyi Zhao, Liying Liu, Lizhi Cao

Int J Mol Med 29: 65-72, 2012; DOI: 10.3892/ijmm.2011.806

Following the publication of this paper, it was drawn to the Editor's attention by a concerned reader that, for the western blots shown in Fig. 4C, the two left lanes in the 'Fibrillarin' gel slice appeared to be strikingly similar to the mirrored two right lanes in the 'Actin' gel slice, albeit the orientations of the blots were horizontally reversed, such that data which were intended to have shown the results of differently performed experiments appeared to have been derived from the same original source. Moreover, the control β-actin blots featured in Fig. 5A of the above paper were strikingly similar to control blots used in a different context that appeared in a subsequently published paper featuring some of the same authors in *American Journal of Cancer Research*. The authors were contacted by the Editorial Office to offer an explanation for the apparent anomaly in the presentation of the data in this paper, although up to this time, no response from them has been forthcoming. Owing to the fact that the Editorial Office has been made aware of potential issues surrounding the scientific integrity of this paper, we are issuing an Expression of Concern to notify readers of this potential problem while the Editorial Office continues to investigate this matter further.

